# A genome-wide survey for SNPs altering microRNA seed sites identifies functional candidates in GWAS

**DOI:** 10.1186/1471-2164-12-504

**Published:** 2011-10-13

**Authors:** Kris Richardson, Chao-Qiang Lai, Laurence D Parnell, Yu-Chi Lee, Jose M Ordovas

**Affiliations:** 1 Nutrition and Genomics Laboratory, Jean Mayer United States Department of Agriculture Human Nutrition Research Center on Aging at Tufts University, Boston, MA, USA; 2 Department of Cardiovascular Epidemiology and Population Genetics, Centro Nacional de Investigaciones Cardiovasculares (CNIC), Madrid, Spain; 3 Instituto Madrileño de Estudios Avanzados (IMDEA) Alimentacion, Madrid, Spain

## Abstract

**Background:**

Gene variants within regulatory regions are thought to be major contributors of the variation of complex traits/diseases. Genome wide association studies (GWAS), have identified scores of genetic variants that appear to contribute to human disease risk. However, most of these variants do not appear to be functional. Thus, the significance of the association may be brought up by still unknown mechanisms or by linkage disequilibrium (LD) with functional polymorphisms. In the present study, focused on functional variants related with the binding of microRNAs (miR), we utilized SNP data, including newly released 1000 Genomes Project data to perform a genome-wide scan of SNPs that abrogate or create miR recognition element (MRE) seed sites (MRESS).

**Results:**

We identified 2723 SNPs disrupting, and 22295 SNPs creating MRESSs. We estimated the percent of SNPs falling within both validated (5%) and predicted conserved MRESSs (3%). We determined 87 of these MRESS SNPs were listed in GWAS association studies, or in strong LD with a GWAS SNP, and may represent the functional variants of identified GWAS SNPs. Furthermore, 39 of these have evidence of co-expression of target mRNA and the predicted miR. We also gathered previously published eQTL data supporting a functional role for four of these SNPs shown to associate with disease phenotypes. Comparison of F_ST _statistics (a measure of population subdivision) for predicted MRESS SNPs against non MRESS SNPs revealed a significantly higher (P = 0.0004) degree of subdivision among MRESS SNPs, suggesting a role for these SNPs in environmentally driven selection.

**Conclusions:**

We have demonstrated the potential of publicly available resources to identify high priority candidate SNPs for functional studies and for disease risk prediction.

## Background

microRNAs (miRs) are small 20-24 nucleotide (nt) noncoding RNAs that mediate translational repression by binding to miR recognition elements (MREs) found in the 3'UTR of their mRNA targets [1]. The most critical region for binding and repression of mRNA by a miR are positions 2-7 of the MRE, referred to as the seed site. Although there are examples of miRs targeting mRNAs without perfect Watson-Crick complementarity to the MRE seed site (MRESS), a collection of evidence supports the MRESS as the most important feature for prediction and function. In some cases single 7mer seed sites are sufficient for a miR to repress translation, and *ex-vivo *experiments have shown single point mutations in the MRESS may reduce effectiveness or abolish miR mediated repression [2]. Further highlighting the importance of this sequence, it has been demonstrated that a higher degree of negative selection occurs within predicted conserved MRESSs compared to conserved non MRESS control sites [3]. Given the importance of the MRESS, it has been proposed that single nucleotide polymorphisms (SNPs) mapping within the MRESS, or which create novel MRESS (CNM), may have functional consequences resulting in phenotypic variation [4]. Moreover, SNPs that create or abrogate MRESSs may modulate gene transcript and protein levels relevant to a phenotype of interest, generally, or under the influence of particular environmental conditions.

It has long been thought that disease causing variants act through alteration of exon sequence resulting in altered protein function. However, SNPs may also act to modulate gene expression, and this has been demonstrated for many promoter SNPs in which the risk allele alters the affinity of a transcription factor to its binding motif [5,6]. Furthermore, several published examples show functional variants in MREs that modulate risk for a variety of disease states, such as breast cancer, Tourette's syndrome, and hypertension among others [4]. Two studies have demonstrated a gene by environment interaction where a MRESS SNP modulates individual response to drug and dietary intakes [7-9]. A survey of the frequency of predicted and validated MRESS SNPs, identified an appreciable number of SNPs falling within MREs across the human genome [10]. However, the number of risk alleles identified with plausible mechanisms for modulation of gene expression is outweighed by SNPs falling in gene desert regions [11]. It could be that these SNPs fall within distant but bona fide enhancer or suppressor elements resulting in the modulation of gene expression, as was demonstrated for the variant within the 8q24 gene desert and its effects on *TP53 *expression in prostate [12]. Alternatively, it may be these SNPs are in LD with variants not yet identified or available on GWAS chips. For example, sequencing of the *HLA-C *3'UTR revealed a SNP modulating an MRE for the binding of miR-148. Furthermore, this SNP was shown to be in LD with rs9264942 which is found 35 kb upstream of *HLA-C *and associates with control of HIV [13]. These data demonstrated that rs9264942 is a marker for a functional SNP that was not contained in commercial SNP arrays. Further underscoring this point, recent chromatin studies have identified novel non-coding gene regulatory regions, some of which contain top scoring hits for disease associating SNPs [14].

Currently over 1000 human miR sequences are reported in the miRbase catalog [15]. Estimates suggest that over 30% of human protein-coding genes are regulated by miRs, and that each miR may potentially regulate hundreds of target transcripts [16,17]. Given this large number of potential miR targets in the human genome, identifying allele-specific miR-mRNA interactions may help elucidate functional roles for a portion of the many SNPs identified in genome wide association studies (GWAS) that lack obvious functionality.

With such information in mind, one aim of the 1000 Genomes Project is to catalog over 95% of human variation in order to inform association studies of all potential causal SNPs [18]. Furthermore, initial studies in the 1000 Genomes pilot indicated that a substantial number of variants are in LD with known disease markers and that these variants are not well covered on commercial arrays. Importantly, the data currently available in the 1000 Genomes Project provides unprecedented access to millions of SNPs, some of which may elucidate functional mechanisms for the many risk alleles identified in GWAS.

Here we have performed a genome-wide survey for SNPs falling within both experimentally validated and computationally predicted conserved MRESSs, by utilizing these data (dbSNP build132) [18]. In addition to this analysis, we have surveyed these data for predicted CNM SNPs. Furthermore, we have examined all SNPs identified in GWAS for functional variants in relation to predicted MRESSs and CNM SNPs using the data from the 1000 Genomes Project. Combing with several other publically available data sources, we identified numerous MRESS SNPs as possible modulators of disease relevant phenotypes. Our work demonstrates the utility of the data generated from the 1000 Genomes Project and provides insight into the frequency and relevance of MRE SNPs in human disease and may provide some clues regarding environmentally driven human selection.

## Results and Discussion

### Approximately 5% of validated MREs contain SNPs in their seed site

To assess the frequency of SNPs falling in validated MRESSs, we first determined the genomic DNA (gDNA) coordinates of 606 validated mRNA target seed-sites for all mRNA-miR interactions, from the miRecords database [19]. For a site to be included in this list we required functional evidence for the target site (eg, loss of function experiment through a reporter assay system). We searched each reported validated site for 4 classes of "canonical" seed sites. Here we define canonical seed sites as having, at least, perfect pairing among seed site positions 2-7 (6-mer) in addition to three other classes with binding site characteristics at positions 1 or 8, demonstrated to improve likelihood of repression; 8mer (an A nt at position 1, and a complementary nt at position 8), 7mer-8m (complementary nt at position 8), and 7-mer-A1 (an A nt at position 1) [1]. We then determined if the gDNA coordinates of all 3'UTR SNPs (from dbSNP132) fell within the gDNA coordinates of each validated MRESS from above. We identified 31 SNPs (5%) that lie in validated MRESSs corresponding to 28 target transcripts (Table 1).

**Table 1:** SNPs found within validated MRESSs.

Rs#	Coordinates	Maj	Min	MAF	Site type	Pos in MRE	miR	Gene	pubmed id
rs3783620	1: 101204463	G	A	.005 (YRI.P1)	8-mer	7th	hsa-miR-126	VCAM1	18227515
rs1059479	1: 113243892	T	G	.01 (CEPH)	8-mer	1st	hsa-miR-138	RHOC	20232393
rs12392	2: 198351529	G	A	NA	7-8mer	2nd	hsa-miR-1	HSPD1	17715156
rs5186*	3: 148459988	A	C	.306 (CEU.P1)	7-8mer	4th	hsa-miR-155	AGTR1	16675453
rs56109847*	3: 183824557	G	A	.992 (CEU.P1-LC)	8-mer	4th	hsa-miR-510	HTR3E	18614545
rs3731563	3: 48199695	T	C	.017 (GIH)	8-mer	8th	hsa-miR-21	CDC25A	19826040
rs1434536*	4: 96075965	C	T	.545 (TSC-CSHL)	7-8mer	1st	hsa-miR-125b	BMPR1B	19738052
rs6875894	5: 112179965	C	T	.027 (YRI)	7-8mer	4th	hsa-miR-135b	APC	18632633
rs6875894	5: 112179965	C	T	.027 (YRI)	7-8mer	5th	hsa-miR-135a	APC	18632633
rs79468771	6: 135539805	T	A	NA	8-mer	7th	hsa-miR-15a	MYB	18818396
rs33986155	6: 152420685	C	G	.083 (CEU.P1)	7-8mer	8th	hsa-miR-206	ESR1	17312270
rs11551509	6: 34505633	C	A	NA	8-mer	8th	hsa-miR-510	SPDEF	18922924
rs8829	7: 148504618	A	C	1 (CEPH)	8-mer	2nd	hsa-miR-101	EZH2	20478051
rs78899540	7: 27181092	A	C	.04 (YRI.P1)	7-mer-A1	2nd	hsa-miR-130a	HOXA5	17957028
rs117556949	7: 27194074	T	C	.992 (CEU.P1-LC)	8-mer	8th	hsa-miR-196a	HOXA7	15105502
rs12720208*	8: 16850399	G	A	.125 (CEU.P1)	8-mer	6th	hsa-miR-433	FGF20	18252210
rs78202059	8: 26228382	G	T	.144 (YRI.P1-LC)	7-mer-A1	8th	hsa-miR-222	PPP2R2A	20103675
rs1058153	2: 46987391	C	T	NA	8-mer	2nd	hsa-miR-21	SOCS5	17991735
rs72808106	10: 74035161	A	G	NA	7-mer-A1	4th	hsa-miR-221	DDIT4	20018759
rs16917496*	12: 123893830	C	T	.22 (CEPH)	7-mer-A1	2nd	hsa-miR-502-5p	SET8	19789321
rs111842797*	12: 123893831	A	G	NA	7-mer-A1	8th	hsa-miR-502-5p	SET8	19789321
rs76290581	12: 6760093	T	C	.051 (YRI.P1-LC)	8-mer	1st	hsa-miR-650	ING4	20381459
rs77080081	15: 40382144	A	G	.014 (CEU.P1)	7-8mer	1st	hsa-miR-125b	BMF	19471102
rs28521337*	15: 88521280	C	G	.467 (CEU.P1)	8-mer	8th	hsa-miR-485-3p	NTRK3	19370765
rs72481816	15: 88521572	G	C	NA	8-mer	8th	hsa-miR-765	NTRK3	19370765
rs72481814	15: 88522372	T	C	NA	7-8mer	4th	hsa-miR-509-3p	NTRK3	19370765
rs28574753	16: 28109760	G	A	.076 (YRI-P1)	7-8mer	2nd	hsa-miR-122	XPO6	19296470
rs75817141	17: 12044581	C	T	.02 (YRI.P1)	7-8mer	1st	hsa-miR-15b	MKK4	19861690
rs62062994	17: 48261978	G	T	NA	8-mer	3rd	hsa-miR-29c	COL1A1	18390668
rs3218074	19: 30315176	A	G	.01 (PDR90)	8-mer	8th	hsa-miR-15b	CCNE1	18701644
rs3218074	19: 30315176	A	G	.01 (PDR90)	8-mer	8th	hsa-miR-16	CCNE1	18701644
rs6094029	20: 43356176	C	T	0	7-8mer	7th	hsa-miR-449a	WISP2	19351815
rs78301106	20: 62522710	C	G	.045 (CHB+JPT.P1)	8-mer	7th	hsa-miR-122	TPD52L2	19296470

A list comprising 31 validated MREs (with perfect complimentarity in the MRE seed site -positions 2-7) in which a SNP has been identified. The Coordinates column provides the chromosomal number and position coordinates for each SNPs - all genomic coordinates correspond to Hg19. The MAF column reports the allele frequency of the minor allele from the population where it was highest, where NA indicates a SNP with unknown allele frequency. The Pos in MRE column refers to the position within the MRE to which the SNP maps. * SNPs reported to have shown association with disease phenotypes.

No population frequency data are available for 29% of the MRE SNPs (9 of 31), a value that will change as whole genome sequence data from more individuals surface and with completion of more encompassing GWAS studies. Nine SNPs have minor allele frequencies (MAF) < = 2%, and may be considered rare in the general population and therefore unlikely as common factors in complex disease. The 13 remaining SNPs have allele frequencies above 2% in at least one population listed in dbSNP (Table 1). Of note, 7 of the 31 (22%) SNPS identified have shown association with disease traits, emphasizing the potential importance of MRESS SNPs as modulators of disease risk.

**Figure 1:**
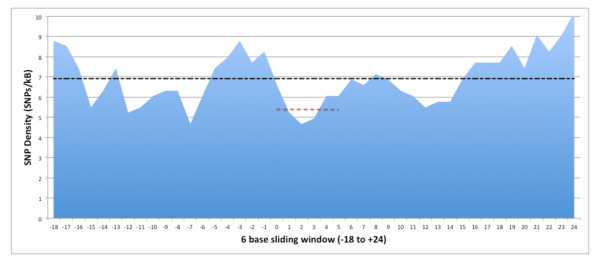
**A measure of SNP density (SNPs/kb) generated from the analysis of a 6 base window sliding over a 42-base region - centered on the first position of the seed site - of 606 validated MREs**. The black line indicates the number of SNPs/kB across the 42 base region. The red line indicates the average SNP density across the 6 windows of seed positions 2-7.

Previous studies have estimated MRESS SNP density to be lower than that observed in regions outside the MRESS, suggesting a higher rate of negative selection on MRESS [3,10]. In light of the updated account of variation in the human genome available in dbSNP build132, we estimated the frequency of SNPs falling within MRESS and those falling outside of MRESSs using the 606 sequences from validated target sites identified from the above analysis. We performed a sliding window search of 6 bases, (the size of seed positions 2-7) starting 18 bases upstream and continuing to 24 bases downstream of each validated MRE site, sliding at a 1 base step. The 0 mark of the x-axis in Figure 1 demarcates the second position of the MRESS (or first position of the 6mer seed site). Our data indicate that the MRESS contains the lowest amount of variation across the region, an observation in agreement with prior analyses [3,10]. Although there is less variation across the MRESSs there appears a considerable density of SNPs (5.5/kb) falling within what is thought to be the most important sequence for miR-mRNA interactions.

### Genome wide survey of predicted MRESS and CNM SNPs

Although many variants have been associated with the modulation of phenotypes relevant to disease in GWAS studies, the challenge of determining which of them may be casual remains [20]. To identify potential causal variants mediated by MRESS creation or disruption we first performed a genome-wide survey for SNPs falling within computationally predicted conserved MRESSs. To do this, we utilized the http://microRNA.org portal to access a collection of predicted miR-mRNA interactions. These predictions were derived using an algorithm that incorporates an array of the most recent miR prediction guidelines, such as seed-site pairing, site context, free-energy, and target conservation across multiple vertebrates [21]. We focused our analysis on MRESSs conserved across mammals as these are more likely to be of functional significance (see methods) [16]. By comparing the gDNA coordinates of each predicted MRESS against the gDNA coordinates of dbSNP132 SNPs, we identified 2723 MRESS SNPs interrupting 5797 conserved predicted interactions. To further prioritize these hits, we classified them by the type of seed match the MRESS SNP was predicted to interrupt; 8mer (2245), 7mer-8m (3251), 7-mer-A1 (180) or 6mer (121). Although there is overlap in the degree of efficiency of repression by these different seed type classes (likely dependent of site sequence context), there remains a hierarchy with 8mer sites being most efficient [1]. Interestingly 38% (2245) of MRESS SNPs fall within predicted 8mer MRESSs. It has been estimated that ~50% of predicted MREs are potentially functional and it is likely that a portion of the SNPs identified here fall within bona fide MREs [22]. Overall, we estimate that 3% of high confidence predicted conserved MRESSs contain SNPs.

In addition to SNPs that may interrupt MRESSs, SNP alleles may also create MRESSs. To identify potential CNM SNPs we performed a genome-wide computational survey for predicted MRESSs that are created when the mRNA sequence contains the non-reference allele of hg19. Using the Ensembl variation API tools we retrieved the flanking 22 nt sequence from both upstream and downstream of the non-reference allele of every 3'UTR dbSNP132 SNP. Each sequence containing the non-reference allele was analyzed for potential miR-target sites using the miRanda software [23]. This analysis provided us with 22295 CNM SNP creating 49047 miR-mRNA predictions which where also categorized by the seed-type they created; 8mer (10333), 7mer-8m (36188), and 7-mer-A1 (526) or 6mer (2000). It should be noted that there are many more predictions for CNM than MRESS SNPs. This is due to the fact that no conservation constraint was imposed on the CNM SNP predictions (See methods). Considering that many CNM SNPs presumably arise to create new regulatory sites, filtering our hits on conservation status would be counter-intuitive. Of note, we found that approximately 28% (6946/25018) of predicted MRESS and CNM SNPs identified here were first identified by the 1000 Genomes project.

### Some SNPs identified in GWAS are in LD with predicted conserved MRESS and CNM SNPs

GWAS have been a powerful approach to identify genetic variants that contribute to disease risk. However, a functional role for many of the SNPs identified has not been elucidated. It is likely some of these SNPs are in strong LD with unknown functional ones, some of which could be among the predicted conserved MRESS and CNM SNPs. To investigate this possibility, we searched the resulting MRESS and CNM SNP data for variants in LD with SNPs showing association, of GWAS significance, with disease traits and related phenotypes. To do so, we retrieved a dataset of 4817 reported associations, collected from GWAS studies, between 3943 unique SNPs and disease traits shown to have P-values meeting a threshold of < 1.0 × 10-5 [24]. These data were processed through SNAP http://www.broadinstitute.org/mpg/snap/ldsearch.php which yielded a list of SNPs (including those from the pilot 1000 Genomes Project data) in LD with those reported in the GWAS. We limited our search to an r^2 ^of > 0.8 for the CEU population. The results of this query were searched against both the MRESS SNP and the CNM SNP predictions. This query identified 35 instances of an MRESS SNP (some MRESS SNPs are in LD with more than one reported GWAS SNP associating with multiple phenotypes), in LD (r^2 ^> 0.8) with a least one reported GWAS SNP or an original GWAS SNP, associating with disease phenotypes (Additional file 1). In total there were 14 MRESS SNPs in 11 genes associating with 16 traits. We also identified 124 instances of a GWAS SNP that associates with disease traits and is in LD (r^2 ^> 0.8) with CNM SNPs (Additional file 2). There were 73 CNM SNPs in 73 genes associating with 52 traits.

In total we identified 87 SNPs (14 MRESS and 73 CNM SNPs) in very strong LD with SNPs reported as associating with disease related phenotypes. These 87 SNPs represent 2.22% of the total unique SNPs reported in the GWAS data. Using the SNAP pairwise LD tool we determined that 6 of the 3943 GWAS SNPs are in LD with each other, giving us 3940 SNPs or regions associating with disease traits. To determine the possibility of this number occurring by chance we first filtered dbSNP 132 for SNPs having a minor allele frequency (MAF) > = 1%, which was the lowest MAF reported in the GWAS data. We next selected randomly 3940 SNPs and, as we did with the GWAS data, ran them through SNAP to determine the SNPs in LD. From this list we determined the number of SNPs found in our MRESS and CNM SNP data, and this analysis was repeated 1000 times. From these 1000 simulations the mean number of SNPs found in the MRESS and CNM SNP data was 24.99 and the standard deviation 6.19. The probability of finding 87 SNPs by chance based on this distribution was calculated to be 1.08 × 10^-23^. These SNPs may be considered the likely putative functional variants which represent proxy SNPs identified by GWAS.

### Co-expression data identify functional candidates

Support for a prediction of a miR regulating an mRNA target is strongly lent by co-expression of both RNAs. Therefore, to further refine this list of 87 cases where a SNP is predicted to create or abrogate a MRESS and be in LD with a GWAS SNP, we searched for evidence of co-expression of the miR and mRNA using the mimiR web tool [25]. In addition, we also searched the biomedical literature using the PubMed database with the terms of miR name and "expression." To search for mRNA expression in the cognate tissue we queried the NCBI Geoprofiles. These queries revealed miR-mRNA co-expression evidence for 39 of the 87 SNP predictions consisting of 12 MRESS SNPs and 27 CNM SNPs. Table 2 and Additional file 3 indicate the number of tissues in which there is evidence for co-expression between miR and mRNA for which the SNP is predicted to modulate an interaction.

**Table 2:** MRESS SNPs in LD with GWAS variants and showing co-expression of miR and mRNA.

GWAS SNP	P-value	Phenotype	PID	LD	PhastCon	Proxy	Maf	FST	Gene	miR	Allele	SVR	S-T	Co	eQTL
rs10089	2.00E-06	Ileal carcinoids	21139019	1	0.6692	rs10089	0.35	0.08	SLC12A2	hsa-miR-421	C/T	-0.682	8mer	23	NA
rs6504340	6.00E-07	Primary tooth dev	20195514	0.9	0.6635	rs1042822	0.18	NA	HOXB2	hsa-miR-186	G/T	-1.341	8mer	62	NA
rs328	9.00E-23	HDL cholesterol	18193044	0.93	0.685	rs1059611	0.13	0.03	LPL	hsa-miR-136	T/C	-0.635	7mer-m8	36	NA
rs10503669	4.00E-19		18193043	0.93											
rs12678919	2.00E-34		19060906	0.93											
rs17482753	3.00E-11		20031538	0.93											
rs325	8.00E-26		20864672												
rs6590330	2.00E-25	Systemic lupus erythematosus	19838193	1	0.8332	rs1128334	0.06	0.44	ETS1	hsa-miR-381	C/T	-1.166	7mer-m8	33	F
rs1128334	2.00E-11		20169177												
rs10941694	9.00E-06	Chronic kidney disease and serum creatinine concentration	20686651	1	0.7345	rs12522910	0.14	NA	HCN1	hsa-miR-653	T/C	-1.352	8mer	9	NA
rs326	5.00E-12	Triglycerides	18193046	0.96	0.6217	rs13702	0.14	0.42	LPL	hsa-miR-410	T/C	-1.159	8mer	19	NA
rs2083637	2.00E-10	Metabolic Syndrome	20694148	0.92											
rs10105606	4.00E-26	Hypertriglycerdemia	20864672	0.82											
rs1008953	1.00E-07	Psoriasis	20953189	1	0.651	rs2245717	0.86	0.4	SYS1	hsa-miR-150	T/G	-0.704	8mer	49	L, F
rs1443512	6.00E-16	Waist-hip ratio	20935629	0.81	0.581	rs4759058	0.78		HOXC13	hsa-miR-503	C/A	-0.755	7mer-m8	29	NS
rs504963	2.00E-08	Crohn's disease	20570966	1	0.5968	rs485073	0.63	NA	FUT2	hsa-miR-186	A/G	-1.181	7mer-m8	63	NA
rs281379	7.00E-12		21102463	0.9											
rs504963	2.00E-08	Crohn's disease	20570966	1	0.5968	rs603985	0.63	NA	FUT2	hsa-miR-186	T/C	-1.181	7mer-m8	63	NA
rs281379	7.00E-12		21102463	0.9											
rs10923931	4.00E-08	Type 2 diabetes	18372903	1	0.5784	rs835576	0.07		NOTCH2	hsa-miR-218	T/C	-0.718	8mer	31	F, L
rs1295686	1.00E-07	Asthma	20860503	0.96	0.581	rs847	0.76	0.31	IL13	hsa-miR-381	T/C	-1.159	7mer-m8	33	F
rs20541	5.00E-15	Psoriasis	19169254	0.96											

All minor allele frequencies (MAF) reported are for the CEU pilot panel of the 1000 Genomes Project, except where indicted. Abbreviations: PID = PubMed accession, SVR = miRSVR score, PhastCon = conservation score, S-T = seed type. Co = The number of cell and tissue samples in the mimiRNA database for which co-expression of miR and mRNA were found. eQTL = Reports available eQTL data in the mUTHER study, where F = -Fat cell biopsy (n = 160), L = LCL cells (n = 166), and S = skin cell biopsy (n = 160).

### eQTL data support several MRE target predictions when a MRESS or CNM SNP is present

Variation in gene transcript levels is thought to be an important modulator of disease risk in humans and SNPs that may mediate this variation are thought to be of great functional significance [26]. To investigate the contribution of SNPs associating with disease traits to transcript level variation, a number of expression Quantitative Trait Loci (eQTL) studies have been performed [27-29]. These results have demonstrated a number of SNPs associating significantly with expression differences across collected tissue samples. Importantly, these studies have noted differences in the amount of transcript variation across tissue samples, suggesting SNPs may modulate regulatory mechanisms, in some cases, in a tissue specific manner [30]. Interestingly, a recent study has estimated that > 80% of miRs act to lower mRNA levels demonstrating mRNA destabilization is the primary mode of action of miRs on target mRNAs [31].

**Figure 2:**
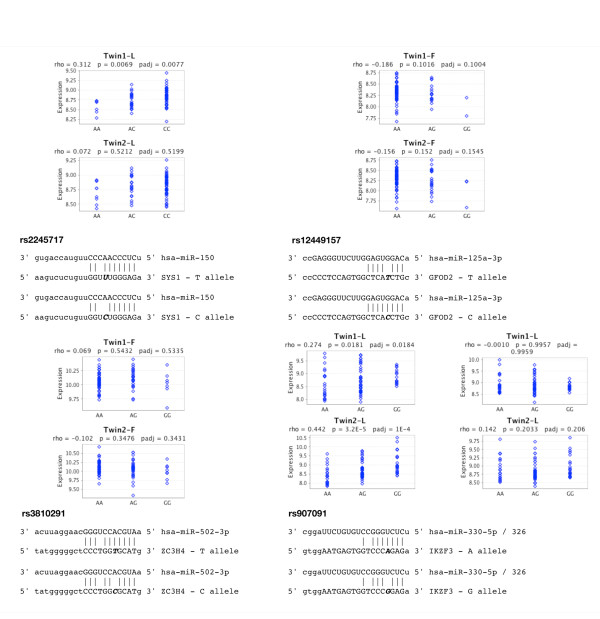
**Four SNPs found to associate, or be in LD with a SNP that associates, with a trait(s) relevant to disease**. Each panel depicts the mRNA-miR interaction and the effect of the SNP on this interaction. Plots were generated using the Genvar web tool and published expression data from Nica, et al. rho = correlation between genotype and transcript levels. p = t = test statistic for correlation. padj = adjusted pvalue for correlation. F = -Fat cell biopsy (n = 160), L = LCL cells (n = 166), and S = skin cell biopsy (n = 160). Twin1 = Unrelated twin group 1. Twin2 = unrelated twin group 2.

To determine if the 39 miR predictions with co-expression data identified in the previous section are supported by eQTL data we utilized the Genevar eQTL database web tool [32]. Genevar allows for querying and visualization of eQTL data for loci of interest using data from various studies. We utilized the results from the recent MuTHER study which reports eQTL data from twin pairs in 3 tissue types; 78 twin-pair lymphoblastoid cell line (LCLs) biopsies, 80 twin-pair skin cell biopsies and 83 twin-pair fat cell biopsies [29]. Searching for eQTL data on each of the 39 MRESS and CNM SNPs we found 11 of the 39 had genotype specific transcript level data in at least one of the 3 tissue samples investigated in the MuTHER study for which there was also evidence of co-expression in mimiR for this tissue. Four of these 11 SNPs showed marginally significant trends in the differences in transcript levels across genotypes (Figure 2).

*SYS1 *transcript levels were shown to be significantly different among rs2245717 genotypes from LCL in one of the two twin study groups (Figure 2). Although, the second twin group failed to achieve significance, the direction of the effect was in agreement with the first group. Furthermore, the lower transcript levels associate with the allele predicted to create the miR-150 binding site. Neither of the two twin adipose tissue sample groups for *GFOD2 *transcript levels showed a significant difference among rs12449157 genotypes. Interestingly, both p-values are of nominal significance and the direction of the effect supports the predicted miR interaction and subsequent effect of the CNM SNP (Figure 2). *IKZF3 *transcript levels measured in LCL cells showed significant differences among the rs907091 genotypes in both twin groups. Lower *IKZF3 *levels were observed in carriers of the G allele which is predicted to create a miR-326 MRESS. However, a second probe found on the Illumina whole genome expression array, used in the study, shows conflicting data where there is no difference between transcript levels in either group. The rs3810291 SNP in the *ZC3H4 *3'UTR shows no significant difference in transcript levels between alleles in adipose samples used in the MuTHER study. However, literature mining for "eQTL, " and the corresponding gene and phenotype identified an additional study showing eQTL data that supports an allelic difference, in the correct direction, and in adipose tissue for rs3810291 [33]. Taken together, this information suggests these four SNPs may have functional significance.

### MRESS and CNM SNPs in Positive Selection

**Figure 3:**
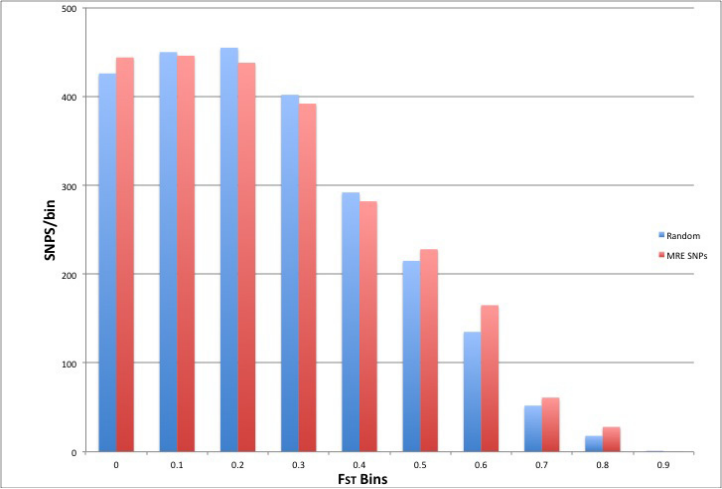
**A plot showing the number of combined MRESS and CNM SNPs or non MRESS and non CNM 3'UTR SNPs across 10 F_ST _bins**. Data plotted compares 2448 MRESS and CNM SNPs with F_ST _data and a random sample of 2448 F_ST _values from the remainder of 3'UTR SNPs. A significant difference between mean FST values for combined MRESS and CNM SNPs and FST values for the remaining 3'UTR SNPs was observed (P = 0.0004).

Genetic variants that have been subject to selection are most likely the functional variants [34,35]. The fixation index (F_ST_) statistic measures population differentiation and provides a test for the influence of selective pressures, where higher F_ST _values indicate local positive adaptation and lower values negative or neutral selection [36]. As adaptive genetic variants have been driven to higher frequencies by environmental factors (i.e, positive selection), SNPs showing high F_ST _values may be considered high priority candidates for association studies for gene by environment studies. Such variants also may play a role in the observed variation and potentially influence disease prevalence across populations [37,38]. To determine if the identified 3'UTR SNPs that create or disrupt predicted MRESSs may be under positive selection we first downloaded genome wide F_ST _calculations for HapMap Phase 3 data [39]. We found a significant difference (P = 0.0004) between the mean transformed F_ST _values of combined MRESS and CNM SNPs (n = 2448) and the remaining (i.e. non-MRESS or non-CNM) 3'UTR SNPs (n = 19906) for which F_ST _data were available. Figure 3 shows the number of F_ST _values between MRESS and CNM SNPs and non-MRE SNPs across 10 F_ST _bins. As F_ST _values increase there is a clear increase in MRESS and CNM SNPs compared to the remainder. This observation further supports that MRESS and CNM SNPs are likely functional variants.

To identify F_ST _outliers we selected all SNPs falling 2 standard deviations (SDs) or more from the mean (Table 3). In total, 24 MRESS or CNM SNPs were identified falling 2 SDs from the mean. Among these is the *GFOD2 *SNP, rs12449157 (F_ST _= 0.8399) for which we show evidence of co-expression and eQTL effect.

**Table 3:** MRESS and CNM SNPs showing highest levels of population sub-division.

MRESS SNPs	FST	Gene	miR	CNM SNPs	FST	Gene	miR
rs3822506	0.8743	TCERG1	miR-590	rs7665492	0.8942	ENAM	miR-3916
rs1217382	0.8469	BCL2L15	miR-17	rs1043809	0.8900	EPN2	miR-3616-3p
rs3087542	0.8428	EMCN	miR-197	rs2470102	0.8859	MYEF2	miR-1180
rs3742988	0.8385	CDAN1	miR-378	rs7290134	0.8695	TNFRSF13C	miR-1205
rs1071738	0.8298	PALLD	miR-182	rs8057598	0.8596	NOL3	miR-769-3p
				rs1969589	0.8545	RGMA	miR-593*
				rs1246014	0.8476	COPS7B	miR-1273d
				rs12449157	0.8399	*GFOD2*	miR-125a-3p
				rs16990309	0.8398	SLC23A2	miR-760
				rs3742988	0.8385	CDAN1	miR-326
				rs2292549	0.8361	GPBAR1	miR-936
				rs1995939	0.8338	STARD9	miR-3943
				rs3199486	0.8321	STARD9	miR-2278
				rs873258	0.8312	TSPAN14	miR-873

MRESS and CNM SNPs showing highest levels of population sub-division among HapMap phase 3 data- All SNPs falling 2 SDs from the mean F_ST _of 3'UTR SNPs.

## Conclusions

In the work presented here, we utilized the latest release of dbSNP, including the 1000 Genomes Project data, to perform a genome-wide scan of human variation within validated and predicted miR binding sites, our hypothesis being that genetic variants at miR binding sites are functional, and important contributors to phenotypic variation and disease susceptibility. We have taken careful measures to assign SNPs as creating or altering miR-mRNA interactions. We identified 5797 instances of a SNP falling within a conserved predicted MRESS based on stringent filtering of conservation and interaction scores predicted by Betel et al [21]. Interestingly, 38% of these predicted disruptions were identified in 8mer target predictions. 8mer target sites have been shown to have the highest efficacy of target repression and therefore are considered higher priority predictions than those with lesser complimentarity [1]. Overall, we estimate that 3% of predicted conserved MRESSs contain SNPs. Our analysis also identified 49407 instances of a SNP creating an MRESS. Given that no conservation restraint was utilized for identification of these CNM SNPs, we must be cautious for it is likely many of them are potential false positives. We also determined that 87 of the MRESS and CNM SNPs identified are in LD with SNPs identified in GWAS. We demonstrate that 2.2% of GWAS SNPs are, or are in LD with, MRESS or CNM SNPs. However, this may be a conservative estimate, given that 1) we limited our SNP selection based on conservation and other strict cutoffs, 2) the catalog of GWAS SNPs investigated is not all encompassing 3) that these GWAS studies do not consider gene by environment interactions and 4) LD estimates only cover SNPs up to the 1000 Genomes Project pilot study 1 data.

Recently, it has been demonstrated that SNPs previously identified in GWAS are in LD with SNPs found in enhancer motifs regulating gene expression [14]. Furthermore, other studies have linked SNPs falling in gene regulatory motifs, and not found on commercial SNP arrays, to be in LD with top scoring GWAS hits [12,13]. In a similar fashion, we suggest that many of the SNPs found in this study to be in LD with GWAS SNPs may have functional significance. To further explore this possibility we utilized several publicly available data sets and tools and showed 39 of these 87 variants found to have evidence of co-expression of target mRNA and the predicted miR. We found that four SNPs from this list have supporting eQTL data demonstrating variation in transcripts between alleles.

Our analyses have identified four SNPs predicted to modulate allele-specific miR-mRNA interactions which are supported by co-expression and eQTL data. The rs907091 SNP falls in the *IZKF3 *transcript and is in LD (r2 > 0.90) with eight SNPs associating with increased risk for a variety of autoimmune diseases. IZKF3 is a transcription factor important for B-cell activation, and mice lacking this gene develop a lupus like syndrome, suggesting a role for IZKF3 in autoimmunity [40]. The rs907091 minor T allele is predicted to create a CNM for mir-326. There is evidence for expression of miR-326 and *IZKF3 *in human B-lymphocytes. Interestingly, miR-326 is important for T-cell differentiation and has been implicated in the pathogenesis of autoimmune multiple sclerosis [41]. A study investigating transcript levels between the T and C alleles of rs907091 in a lymphoblastoid cell line (LCL) demonstrate significantly lower levels of *IZKF3 *in subjects carrying the T allele [29]. These data suggest that carriers of the T allele may have reduced levels of IZKF3, in part through miR-326.

In addition the minor allele of rs3810291 is predicted to create an MRE for mir-502-3p within the *ZC3H4 *transcript and associates with BMI. ZC3H4 is a poorly characterized zinc finger protein. There is eQTL evidence supporting this prediction where minor allele carriers have reduced *ZC3H4 *expression compared to non-carriers, in adipose tissue [33]. Both mir-502-3p and *ZC3H4 *are expressed in adipose [42]. The rs2245717 SNP, predicted to create an MRE for miR-155 in the *SYS1 *transcript, is in perfect LD with rs1008953 a SNP associating with psoriasis [43]. The MRE-creating allele of *SYS1 *is also associated with lower *SYS1 *transcript levels in LCL cells [29]. While a role for SYS1 in immune function could not be found in the literature, it is known that miR-155 is involved in the immune response [44]. The rs12449157 SNP is found in the poorly characterized glucose-fructose oxidoreductase domain containing 2 (*GFOD2*) transcript showing association with HDL-C [45]. Our analysis predicts that the minor allele of rs12449157 creates a CNM for mir-125a-3p and that it is associated with reduced *GFOD2 *levels. Interestingly, both RNAs are expressed in adipose tissue [42]. Further, we identify rs12449157 as an F_ST _outlier suggesting this SNP may be undergoing population specific selection.

In addition to these four SNPs, we identified 39 others with data indicating co-expression with the predicted target mRNA and these should be considered as candidates for functional studies. Of these 39 candidates, a SNP within the *HOXB2 *loci has shown eQTL peaks identified from lymphoblastoid cell lines [28,46]. While our analysis has generated many MRESS and CNM SNP predictions for which no miR expression data are available, it is likely that as more miR expression and eQTL data become accessible, particularly for different cell types and specific conditions, many of these SNPs could be seen as functionally relevant. Recent data indicate some miRs may act intracellularly, carried by HDL particles to recipient cells [47]. Therefore, it may be that co-expression is not essential for all predicted miR-mRNA interactions.

As new variants arise in a population and are exposed to different environmental conditions, those variants may be subject to forces of selection. Moreover, if these SNPs alter gene expression they may modulate the individual's response to the environment and potentially the risk for particular disease state. Based on this, we hypothesized that allele-specific miR-mRNA interactions would show a greater level of selection than SNPs not classified as MRESS SNPs. We show that, as a group, predicted MRESS and CNM SNPs have a significantly higher mean F_ST _than do those SNPs which do not create or disrupt a predicted MRESS. We identify those MRESS and CNM SNPs showing the highest degree of population subdivision and suggest these SNPs and the interactions they are predicted to modulate, as candidates for functional studies.

We show that the frequency of MRESS SNPs in validated MREs (5.5 SNPs/kb) is less than in surrounding regions and this supports prior work showing a higher degree of negative selection on MRESSs [3,10]. Although the level of variation within this region is lower, we do show that the occurrence of variation across validated MRESSs is not rare (~5%). Supporting the notion that miR SNPs are high priority candidates for functional consequence we show that 22% of SNPs falling within validated MRESSs have reported associations related to a disease phenotype or risk. Of note, our results differ somewhat from the MRESS SNPs reported in Saunders, et al [10]. This is most likely due to the fact that we utilized a more current database of validated MRE targets, and also that we required functional evidence of MRESS for inclusion.

There are several web based MRE SNP prediction databases available to query a SNP for creation or disruption of a MRESS, however these tools incorporate a relatively limited amount of functional annotation (GWAS, co-expression and eQTL data) for identification of the most promising MRESS SNPs [48-50]. SNPinfo, is a web tool which offers the calculation of LD between query SNPs and GWAS SNPs in addition to functional prediction of these SNPs for abrogation or creation of potential MRESS [50]. Approximately 70% of the SNPs found in Tables S1 and S2 are also identified at SNPinfo web portal as being a SNP in LD with a GWAS SNP, and SNPinfo also includes prediction of that SNP as a MRESS SNP. Importantly, our work differs from what may be found at SNPinfo and others, in that we present a more comprehensive summary of potential MRESSs SNPs, being the first to investigate 1000 Genomes data for MRESS SNPs. Furthermore, we use this MRESS SNP information in combination with a variety of publically available web tools and data sets (including co-expression and eQTL data), not currently incorporated in other resources, to determine which of these SNPs are most likely functional. Our data demonstrates the utility of using multiple publically available datasets and resources to identify functional candidates.

In Summary, we have surveyed the most current human SNP data and identified variants that provide functional hypotheses for observed GWAS associations. Our work also suggests that a considerable number of SNPs create or abrogate MREs in the human genome. Our results further suggest MRE SNPs that modulate gene expression are likely to be under selective pressure. With relevance to human disease we show that publicly available resources can be used to identify high priority candidate SNPs for functional studies.

## Methods

### Retrieval and use of dbSNP information

We retrieved all dbSNP build 132 SNP (as of 11-31-10) information by downloading the vcf file available through the 1000 genomes home page [18]. To ensure we only surveyed variation in the form of SNPs and not indels and/or copy number variants we removed all SNPs not reported as bi-allelic. A subset of data containing all 3'UTR SNPs (n = 210042) was extracted using Perl. This dataset was used for all subsequent analyses. To determine the percentage of SNPs that were submitted to dbSNP by the 1000 Genomes Project we used UCSC Genome Browser to identify all dbSNP build 132 SNPs where the submitter status handle was equal to only the 1000GENOMES tag. This data set was then searched against our MRESS and CNM data sets to identify those SNPs contributed by the 1000 Genomes Project. To retrieve allele frequency data on SNPs reported in MRESSs and CNMs, we utilized the Perl API variation tools accessing the latest human genome variation data, build 61. All data analysis was performed on the NUGO information network [51].

### Identifying MRE SNPs in validated targets

miRecords hosts a collection of validated miR-mRNA interactions built from an exhaustive literature search and the database of records was download in a tab-delimited format [19]. We next annotated each hit for target site functionality, by checking the literature source for evidence of a loss of function experiment, which provided us with 606 validated MRE targets. To identify SNPs falling within these 606 validated targets sites, a Perl script was written to search each SNP gDNA coordinate against the gDNA coordinates of each target transcript MRESS, retrieved from Ensembl.

### SNP Density determination

To calculate SNP density, a Perl script was written to perform a sliding window search (W = 6 bases as this corresponds in size to bases 2-7 of the MRESS) of the 606 validated MREs for SNPs, starting 18 bases upstream of the first base of the MRESS and ending 18 bases downstream of the last base of the MRESS. We report the number of SNPs for each position of the window across this sequence. Values are reported as SNPs/kb.

### Identification of conserved MRESS and CNM SNPs

To identify SNPs falling in conserved MRESSs, we downloaded the "good mirsrv_score conserved miRNA" datafile from the http://microrna.org website. This file contains all predicted mRNA target motifs for targeting microRNAs which belong to conserved microRNA families. Conservation signal is used to predict functional MREs, however it has been determined that a conservation signal above background for MREs of the most recent mammalian specific miRNA families (non-conserved) was unlikely due to the relatively short time between the emergence of these miRs and the occurrence of new MREs within 3 'UTRs [16]. Therefore, to eliminate false positives that would arise from this form of analysis we utilize predictions for only conserved miR families - which are contained in the "good mirsrv_score conserved miRNA" datafile. To add an additional measure of conservation, we implemented a conservation score cutoff for predicted miR targets using a Phastcon score of > = 0.57, which authors of the work estimate corresponds to conservation across the mammalian genomes used in their study [21,52,53]. The Phastcon scores are provided in the predictions data file from http://microRNA.org. Additionally, predictions generated by the http://microRNA.org tool provide a ranking score (mirSVR score) which is calibrated to correlate linearly with the extent of down regulation of a miR on its target. Importantly, these scores may be interpreted as an empirical probability of down regulation. From these data we selected a mirsvr score cutoff < = -0.6, representing the top 12% of all predictions. A Perl script was then used to compare the gDNA coordinates of each predicted MRESS (n = 197287) against the gDNA coordinates of each 3'UTR SNP in dbSNP132.

To identify CNM SNPs, we utilized the Ensembl variation Perl API tools (Build 61) to retrieve the 22 bases flanking the 5' and 3' regions of every 3'UTR SNP in dbSNP 132. We generated the reverse complement for those mRNAs transcribed from the negative strand. These data were then run locally through the miRanda target prediction algorithm. To limit identification of potential false positives we implemented an arbitrary paring score cutoff of > = 150 and an energy cutoff of < = -20. We identified all predicted MRESS created by CNM SNPs by filtering hits on the position of their target prediction on the mRNA, where each SNP is located at position 23 of 45.

### Retrieval of GWAS results and LD calculations

We first downloaded a catalog of human variation associating with disease phenotypes (1-25-11) [24]. The list was then submitted to the SNAP tool http://www.broadinstitute.org/mpg/snap/ldsearch.php using r^2 ^of > 0.8 for the CEU population. The resulting list was then searched against each of the CNM and MRESS SNP lists to identify 87 MRESS and CNM SNPs. To determine the probability of observing this number by chance, 3940 SNPs were randomly selected from the dbSNP build 132 data set filtered for SNPs with a MAF > = 1% and run through the SNAP tool to identify all SNPS in LD, using r^2 ^of > 0.8 for the CEU population. This procedure was repeated 1000 times. The probability of observing 87 MRESS or CNM SNPs randomly from the genome was determined based on the normal distribution generated from the 1000 simulations.

#### Co-expression

We utilized the mimiRNA web tool to identify miR-mRNA predictions with co-expression evidence [25]. The mimiRNA tool provides expression data for 564 mRNAs and 636 miRs, normalized across samples, from four large scale miR expression studies, and one mRNA expression study. We queried each miR-mRNA pair for co-expression using the tools provided on the webpage. Because not all of the miRs implicated in our work are in the mimiRNA dataset, we also searched the literature via PubMed using the search terms of the miR-name and the term "expression." To determine if the matching gene was expressed in the same tissue type, we queried the GEOprofile database.

#### eQTL survey

To identify association of transcript levels with MRESS and CNM SNPs we searched eQTL data from the MuTHER study using the Genevar web tool [29,32]. eQTL data was generated from Fat cell biopsy (n = 160), LCL cells (n = 166) and skin punch biopsy (n = 160) taken from healthy adult female twins (both mono and di-zygotic). Twin pairs where separated in two to unrelated groups, thereby performing 2 independent eQTL analysis, as described in Nica, et al.

Genevar provides Spearman's rank correlation coefficient estimates for the strength of relationships between alleles and gene expression intensities for each study group. Furthermore, to test the significance of the relationship, Genevar generates a t-statistic for correlation analysis. Adjusted non-parametric permutation P-values are also provided [32].

### F_ST _***calculations***

Genotype characteristics of 11 HapMap Phase 3 populations were split into 4 groups of similar ancestry; Asian, African, European and American. F_ST _values where calculated for each HapMap Phase 3 SNP between these 4 groups and reported in a downloadable file [39]. Using a Perl script we extracted all MRESS and CNM SNPs with F_ST _values from this dataset. We used the statistical analysis software (SAS) boxcoxar macro to transform the F_ST _data to fit a normal distribution. We then performed an unpaired Student's t-test using the transformed values for these two groups to determine if they were significantly different. To identify MRESS and CNM F_ST _outliers we selected those SNPs with F_ST _> 2 SDs from the mean.

Authors have no competing interests to declare.

## Authors' contributions

KR wrote all Perl scripts for comparisons and analysis. KR and YCL performed all genome SNP scans. KR and LDP performed sliding window SNP density analysis. KR and CQL performed F_ST _analysis. KR, CQL, and JMO conceived the work and drafted the manuscript. All authors read and approved the manuscript.

## Supplementary Material

Additional file 1 **SNPs identified to abrogate MRESSs in LD with GWAS SNPs**. All MRESS SNP minor allele frequencies (MAF) reported are for the CEU pilot panel of the 1000 Genomes Project, except where indicted. *indicates MAF in low coverage 1000genomes CEU panel. Abbreviations: GWAS SNP = SNP reported in GWAS, Proxy = MRESS SNP in LD with GWAS SNP, SNP coord = genomic coordinate of MRESS SNP, PhastCon = conservation score, PID = PubMed accession, SVR = miRSVR score, S-T = seed type. Click here for file

Additional file 2 **CNM SNPs in LD with GWAS SNPs**. CNM SNPs in LD with variants association with disease traits. All CNM SNP minor allele frequencies (MAF) reported are for the CEU pilot panel of the 1000 Genomes Project, except where indicted. * indicates MAF in low coverage 1000genomes CEU panel. Abbreviations: GWAS SNP = SNP reported in GWAS, Proxy = CNM SNP in LD with GWAS SNP, SNP coord = genomic coordinate of CNM SNP, PMID = PubMed accession, PS = miRanda Pairing Score, ES = miRanda energy score, S-T = seed type. Click here for file

Additional file 3 **CNM SNPs in LD with GWAS variants and showing co-expression of miR and mRNA**. CNM SNPs in LD with variants association with disease traits. All minor allele frequencies (MAF) reported are for the CEU pilot panel of the 1000 Genomes Project, except where indicted. * indicates MAF in low coverage 1000genomes CEU panel. Abbreviations: PMID = PubMed accession, PS = miRanda Pairing Score, ES = miRanda energy score, S-T = seed type, miRlit = evidence of miR and mRNA expression collected from the literature, where numbers indicate pubmed ids, except those beginning with GDS, which indicate the Geoprofile dataset ID for which expression was demonstrated. Co = The number of cell and tissue samples in the mimiRNA database for which co-expression of miR and mRNA were found. eQTL = Reports available eQTL data in the mUTHER study, where F = -Fat cell biopsy (n = 160), L = LCL cells (n = 166), and S = skin cell biopsy (n = 160). Click here for file
