# Supplementary material for: A genome-wide survey for SNPs altering microRNA seed sites identifies functional candidates in GWAS
Source: BMC Genomics. 2011 Oct 13;12:504. doi: 10.1186/1471-2164-12-504 (PMC3207998; doi:10.1186/1471-2164-12-504)
Supplement: Additional file 3 — CNM SNPs in LD with GWAS variants and showing co-expression of miR and mRNA. CNM SNPs in LD with variants association with disease traits. All minor allele frequencies (MAF) reported are for the CEU pilot panel of the 1000 Genomes Project, except where indicted. * indicates MAF in low coverage 1000genomes CEU panel. Abbreviations: PMID = PubMed accession, PS = miRanda Pairing Score, ES = miRanda energy score, S-T = seed type, miRlit = evidence of miR and mRNA expression collected from the literature, where numbers indicate pubmed ids, except those beginning with GDS, which indicate the Geoprofile dataset ID for which expression was demonstrated. Co = The number of cell and tissue samples in the mimiRNA database for which co-expression of miR and mRNA were found. eQTL = Reports available eQTL data in the mUTHER study, where F = -Fat cell biopsy (n = 160), L = LCL cells (n = 166), and S = skin cell biopsy (n = 160). [file 1471-2164-12-504-S3.DOC]

| **GWAS SNP** | **P-value** | **Phenotype** | **PID** | **LD** | **Proxy** | **Maf** | **FST** | **Gene** | **miR** | **Allele** | **PS** | **ES** | **S-T** | **Exp lit** | **Co** | **eQTL** |
| --- | --- | --- | --- | --- | --- | --- | --- | --- | --- | --- | --- | --- | --- | --- | --- | --- |
| **rs2338104** | 1.00E-10 | HDL cholesterol | 19060906 | 0.96 | **rs1045255** | 0.55 | NA | UBE3B | hsa-miR-452 | G/C | 154 | -21.18 | 7mer-m8 |  | 21 | NA |
| **rs2294008** | 4.00E-11 | Bladder cancer | 20972438 | 1 | **rs1045547** | 0.46 | .038 | PSCA | hsa-miR-597 | T/G | 156 | -20.2 | 8mer |  | 7 | NA |
| **rs4343** | 3.00E-25 | Angiotensin-converting enzyme activity | 20066004 | 0.90 | **rs1055086** | 0.48 | NA | ACE | hsa-miR-373* | A/G | 154 | -20.66 | 7mer-m8 |  | 14 | NA |
| **rs9272535** | 9.00E-08 | Chronic lymphocytic leukemia | 21131588 | 0.81 | **rs1064991** | NA | NA | HLA-DQA1 | hsa-miR-20b* | G/C | 157 | -20.53 | 7mer-m8 |  | 4 | NA |
| **rs4929923** | 1.00E-08 | Menarche (age at onset) | 21102462 | 1 | **rs10769931** | 0.70 | NA | TRIM66 | hsa-miR-92b* | T/C | 159 | -26.21 | 7mer-m8 |  | 2 | NA |
| **rs7631605** | 1.00E-06 | P-tau181p | 20932310 | 0.84 | **rs1133661** | 0.458 | NA | EPM2AIP1 | hsa-miR-665 | A/G | 156 | -25.04 | 8mer |  | 4 | NA |
| **rs12449157** | 2.00E-07 | HDL cholesterol | 20864672 | 1 | **rs12449157** | 0.13 | 0.84 | GFOD2 | miR-125a-3p | A/G | 160 | -22.52 | 7mer-m8 | miR:  0497147  mRNA: GDS3688 | 9 | F, L |
| **rs29941** | 3.00E-09 | Body mass index | 20935630 | 0.89 | **rs14810** | 0.67 | NA | KCTD15 | miR-486-3p | C/G | 150 | -24.48 | 8mer | miR:20651284  mRNA: GDS3615 | 2 | NA |
| **rs2967605** | 1.00E-08 | HDL cholesterol | 19060906 | 0.94 | **rs2241588** | 0.16 |  | RAB11B | hsa-miR-92b* | C/T | 154 | -28.35 | 7mer-m8 |  | 2 | NA |
| **rs1008953** | 1.00E-07 | Psoriasis | 20953189 | 1 | **rs2245717** | 0.86 | 0.40 | SYS1 | hsa-miR-188-3p | T/G | 153 | -24.72 | 7mer-m8 |  | 4 | L |
| **rs1008953** | 1.00E-07 | Psoriasis | 20953189 | 1 | **rs2245717** | 0.86 | 0.40 | SYS1 | hsa-miR-532-3p | T/G | 169 | -28.01 | 8mer |  | 6 | L |
| **rs10838738** | 5.00E-09 | Body mass index | 19079261 | 0.86 | **rs2293577** | 0.625 | 0.11 | SLC39A13 | hsa-miR-665 | C/T | 157 | -23.73 | 8mer |  | 5 | NA |
| **rs13098911** | 3.00E-17 | Celiac disease | 20190752 | 0.91 | **rs3136667** | 0.10 | 0.58 | CCR1 | hsa-miR-608 | A/G | 155 | -29.28 | 6mer |  | 5 | NA |
| **rs3743266** | 8.00E-07 | Menarche (age at onset) | 21102462 | 1 | **rs3743266** | 0.33 | 0.36 | RORA | miR-509-3-5p | T/C | 159 | -21.81 | 8mer | miR:  21436257  mRNA:  21102462 | NA | NA |
| **rs3810291** | 2.00E-12 | Body mass index | 20935630 | 1 | **rs3810291** | 0.65* | 0.67 | ZC3H4 | miR-502-3p | G/A | 152 | -21.47 | 7mer-m8 | miR:  20497147  mRNA:  20935630 | NA | F |
| **rs1007738** | 7.00E-07 | Bone mineral density (hip) | 19079262 | 1 | **rs3829940** | 0.77 | NA | LRP4 | hsa-miR-361-3p | A/G | 152 | -22.32 | 7mer-m8 |  | 5 | NA |
| **rs3914188** | 3.00E-07 | Menarche (age at onset) | 21102462 | 1 | **rs3914188** | 0.72 | NA | ECE2 | hsa-miR-612 | G/C | 159 | -28.94 | 7mer-m8 |  | 9 | NA |
| **rs4788084** | 3.00E-13 | Type 1 diabetes | 19430480 | 0.81 | **rs40837** | 0.44 | .026 | IL27 | hsa-miR-661 | A/G | 155 | -21.15 | 7mer-m8 |  | 6 | NA |
| **rs10935120** | 7.00E-08 | Height | 18391952 | 0.80 | **rs4519744** | 0.60 | NA | KY | hsa-miR-663 | T/C | 151 | -35.16 | 7mer-m8 |  | 14 | NA |
| **rs3820928** | 5.00E-06 | Pulmonary function traits (other) | 17903307 | 0.87 | **rs56324594** | 0.46 | NA | COL4A4 | hsa-miR-339-5p | C/T | 179 | -32.84 | 8mer |  | 10 | NA |
| **rs3995090**  **rs11168048** | 4.00E-09  1.00E-11 | Pulmonary function | 20010834  20010835 | 0.93  0.93 | **rs6580550** | 0.33 | 0.29 | HTR4 | hsa-miR-148a | T/C | 159 | -21.6 | 8mer |  | 52 |  |
| **rs3995090**  **rs11168048** | 4.00E-09  1.00E-11 | Pulmonary function | 20010834  20010835 | 0.93  0.93 | **rs6580550** | 0.33 | 0.29 | HTR4 | hsa-miR-148b | T/C | 159 | -20.05 | 8mer |  | 45 |  |
| **rs3995090**  **rs11168048** | 4.00E-09  1.00E-11 | Pulmonary function | 20010834  20010835 | 0.93  0.93 | **rs6580550** | 0.33 | 0.29 | HTR4 | hsa-miR-152 | T/C | 156 | -22.43 | 8mer |  | 44 |  |
| **rs2814707**  **rs3849942** | 5.00E-11  3.00E-25 | Amyotrophic lateral sclerosis | 20801717  20066004 | 0.90 | **rs700782** | 0.21 | 0.04 | IFNK | hsa-miR-153 | C/T | 166 | -21.58 | 8mer |  | 41 | F, L |
| **rs4794822** | 6.00E-10 | Neutrophil count | 20172861 | 0.89 | **rs7021** | 0.4 | NA | PSMD3 | hsa-miR-214* | T/A | 156 | -21.63 | 8mer |  | 4 | NA |
| **rs2271293** | 8.00E-16 | HDL cholesterol | 19060911 | 1 | **rs72556537** | 0.13 | NA | ACD | miR-147 | A/T | 150 | -22.2 | 7mer-m8 |  | 27 | NA |
| **rs2338104** | 1.00E-10 | HDL cholesterol | 19060906 | 0.90 | **rs877710** | 0.54 | 0.41 | MMAB | hsa-miR-564 | C/G | 154 | -26.44 | 8mer |  | 14 | NA |
| **rs887304** | 8.00E-07 | Non-alcoholic fatty liver disease histology (lobular) | 20708005 | 1 | **rs887304** | 0.69 | 0.35 | EFCAB4B | hsa-miR-323-5p | T/C | 150 | -25.81 | 7mer-m8 |  | 2 | NA |
| **rs2336725** | 1.00E-12 | Height | 20881960 | 0.96 | **rs891368** | 0.56 | 0.57 | RFT1 | hsa-miR-497 | A/G | 162 | -20.44 | 7mer-m8 |  | 39 | F, L |
| **rs7216389**  **rs907092**  **rs9303277**  **rs2872507**  **rs2290400**  **rs8067378** | 9.00E-11  8.00E-06  2.00E-09  9.00E-07  6.00E-13  1.00E-07 | Asthma  Primary biliary cirrhosis  Primary biliary cirrhosis  Rheumatoid arthritis  Type 1 diabetes  Ulcerative colitis | 17611496  19458352  20639880  20453842  19430480  20228799 | 0.97  0.91  0.970.88  0.97  0.97 | **rs907091** | 0.53 | 0.39 | IKZF3 | miR-326 | C/T | 153 | -23.56 | 8mer |  | 39 | F , L |
